# DMSO and Its Role in Differentiation Impact Efficacy of Human Adenovirus (HAdV) Infection in HepaRG Cells

**DOI:** 10.3390/v16040633

**Published:** 2024-04-19

**Authors:** Katharina Hofmann, Samuel Hofmann, Franziska Weigl, Julia Mai, Sabrina Schreiner

**Affiliations:** 1 Institute of Virology, School of Medicine, Technical University of Munich, 80333 München, Germany; 2 Tissue Bank of the German Center for Infection Research (DZIF), Partner Site Heidelberg, Institute of Pathology, University Hospital Heidelberg, 69120 Heidelberg, Germany; katharina.hofmann@med.uni-heidelberg.de (K.H.); 3 Institute of Virology, Hannover Medical School, 30625 Hannover, Germany; 4 Heidelberg University, Medical Faculty Heidelberg, and Center for Pediatric and Adolescent Medicine, Department I, Division of Pediatric Neurology and Metabolic Medicine, University Hospital Heidelberg, 69120 Heidelberg, Germany; samuel.hofmann@med.uni-heidelberg.de; 5 Cluster of Excellence RESIST (Resolving Infection Susceptibility; EXC 2155), Hannover Medical School, 30625 Hannover, Germany; 6 Institute of Virology, Medical Center—University of Freiburg, Faculty of Medicine, University of Freiburg, 79106 Freiburg, Germany; julia.mai@uniklinik-freiburg.de

**Keywords:** human adenovirus, HAdV, DMSO, dimethyl sulfoxide, HepaRG

## Abstract

Differentiated HepaRG cells are popular in vitro cell models for hepatotoxicity studies. Their differentiation is usually supported by the addition of dimethyl sulfoxide (DMSO), an amphipathic solvent widely used in biomedicine, for example, in potential novel therapeutic drugs and cryopreservation of oocytes. Recent studies have demonstrated drastic effects, especially on epigenetics and extracellular matrix composition, induced by DMSO, making its postulated inert character doubtful. In this work, the influence of DMSO and DMSO-mediated modulation of differentiation on human adenovirus (HAdV) infection of HepaRG cells was investigated. We observed an increase in infectivity of HepaRG cells by HAdVs in the presence of 1% DMSO. However, this effect was dependent on the type of medium used for cell cultivation, as cells in William’s E medium showed significantly stronger effects compared with those cultivated in DMEM. Using different DMSO concentrations, we proved that the impact of DMSO on infectability was dose-dependent. Infection of cells with a replication-deficient HAdV type demonstrated that the mode of action of DMSO was based on viral entry rather than on viral replication. Taken together, these results highlight the strong influence of the used cell-culture medium on the performed experiments as well as the impact of DMSO on infectivity of HepaRG cells by HAdVs. As this solvent is widely used in cell culture, those effects must be considered, especially in screening of new antiviral compounds.

## 1. Introduction

The liver has multiple functions in the human body, especially in energetic and xenobiotic metabolisms like drug metabolism. It is therefore prone to drug-induced damage, which is why in vitro screening and models for drug safety are very important in the pharmaceutical industry, and also in the reduction in animal testing. The most common cells found in the liver are hepatocytes. These play a major role in detoxification [1,2,3]. Primary human hepatocytes (PHHs) are the gold standard for hepatotoxicity studies. However, they display some disadvantages like batch-to-batch variations of cell viability, inter-individual differences between donors and a quick decrease in function and infectability [4,5,6,7]. During recent years, differentiated HepaRG cells have become more popular. This cell line is a hepatic progenitor cell line obtained from a hepatitis-C-virus-induced tumor [7]. It is currently the closest to PHHs among in vitro liver cell models, as it shows transcriptomic patterns, physiological functions and enzymatic activities comparable to hepatocytes [4,6,8,9,10].

The differentiation of HepaRG cells is usually induced by dimethyl sulfoxide (DMSO), a solvent widely used in pharmacology, toxicology and cell culture, e.g., for cryopreservation of cells, including oocytes for in vitro fertilization [6,11]. Its amphipathic character leads to dissolvement of poorly soluble polar and non-polar molecules and enhances penetration of topological treatments. It is generally accepted as nontoxic below 10% (*v*/*v*) and expected effects of DMSO are assumed as negligible, which is why applied concentrations are often unreported. On the contrary, DMSO is also used as medicine due to its anti-inflammatory, diuretic, vasodilative and muscle-relaxing effects [11]. It was first applied for differentiation of hepatocytes by Isom et al. to extend the time that rat hepatocytes remained differentiated in vitro [4,12,13]. DMSO maintains levels of hepatic transcription factors, nuclear receptor profiles and expression of cytochrome P450 as well as lipid synthesis and secretion processes. Additionally, it inhibits cell apoptosis by, e.g., inactivation of cleaved initiator caspases [4,14,15,16]. An optimal concentration of 2% DMSO (vol/vol) for differentiation of hepatocytes was reported [12]. The molecular mechanisms of the effect of DMSO on cell differentiation and survival are part of current research. Dubois-Pot-Schneider et al. showed that the addition of 2% DMSO to HepaRG cells led to sequential expression of nuclear receptors as well as epigenetic changes based on post-translational modifications of the N-termini of histones. Additionally, cytoskeleton organization and remodeling of extracellular matrix composition was affected [4]. Effects on DNA methylation status were confirmed in a study by Iwatani et al., where hypo- as well as hypermethylation was observed after DMSO treatment (0.1% vol/vol) in the mouse embryoid body, leading to phenotypic changes [17]. Verheijen et al. demonstrated drastic and tissue-specific changes in human cellular processes and epigenetic landscapes in 3D cardiac and hepatic microtissues after treatment with 0.1% DMSO, and concluded that DMSO is not inert, as it is often postulated [11].

DMSO is typically used as a solvent for screening and testing of potential novel therapeutic compounds, including antivirals. Human adenoviruses (HAdVs) are non-enveloped viruses containing a double stranded DNA genome. This large species, with currently 113 HAdV types, can cause different infections in the respiratory tract, the gastrointestinal tract, the renal system and the eye [18,19,20,21]. In immunocompetent individuals, HAdV infections are usually subclinical and self-limiting [22]. In immunocompromised patients like hematopoietic stem-cell transplant recipients, however, they can lead to severe complications and even death [23,24]. Until now, no specific treatment or efficient vaccination has been available, making the screening for an effective and secure antiviral against HAdVs an important and urgent challenge [25].

In this work, infection studies of HepaRG cells with adenovirus were performed, using different concentrations of DMSO from two manufacturers to analyze the impact of DMSO on infectability of HepaRG cells. As recently published, HepaRG cells are an appropriate model system to study adenoviral infection [26]. Additionally, two cell-culture media were compared in this context, namely the widely used Dulbecco’s modified Eagle medium (DMEM) and William’s E medium, which is commonly used for hepatocytes. 

## 2. Materials and Methods

### 2.1. Cell Culture

HepaRG cells [27] (Thermo Scientific, Waltham, MA, USA) were grown either in Dulbecco’s modified Eagle medium (DMEM) or William’s E medium (WE) (both Sigma-Aldrich, St. Louis, MO, USA) at 37 °C and 5% CO_2_. In both cases media were supplemented with 10% fetal bovine serum (FBS), 100 U/mL of penicillin and 100 µg/mL of streptomycin (all three Thermo Scientific), 5 µg/mL bovine insulin and 0.5 µM hydrocortisone (both Sigma-Aldrich). For WE medium, 2 mM of L-glutamine (Thermo Scientific) was added. Cells were cultivated for two weeks before differentiation for another two weeks by addition of different DMSO (SERWA/Carl Roth) concentrations between 0 and 1% with medium changes every two to three days and were tested frequently for mycoplasma contamination.

### 2.2. Viruses and Viral Infection

The used viruses were (1) a replication-deficient, eGFP-expressing HAdV-C5-based first-generation adenoviral vector and (2) a replication-competent HAdV-C5 deltaE3 virus, encoding a CMV-promoter-driven eGFP expression cassette [28]. Viruses were propagated and titrated in HEK293 cells. For this, infected cells were harvested 48 to 72 h post infection (p.i.) and lysed by freezing and thawing cells three times. Subsequently, the supernatant was used to reinfect HEK293 cells. Viral growth was determined by immunofluorescence staining of the adenoviral DNA-binding protein E2A.

HepaRG cells were infected after two weeks of differentiation with/without DMSO with an MOI of 20 for 1 h. Then, 24 h p.i., the number of infected cells was determined by counting eGFP-expressing fluorescent cells at 488 nm wavelength in five fields of view per sample using a Zeiss Axio Observer Z1 and the *Axiovision* software version 3.4.2 (both Carl Zeiss, Oberkochen, Germany).

### 2.3. Cytotoxicity and Cell Viability Assays

Cell viability in response to infection and DMSO treatment was determined (24 h p.i.) using the Promega CellTiter-Blue^®^ (CTB) Cell Viability Assay system, according to the manufacturer’s manual. Fluorescence values were detected using a Promega GloMax^®^ Discover System.

### 2.4. Antibodies and Protein Analysis

Primary antibodies specific for adenoviral proteins used in this study were E2A-72K mouse mAb B6-8 [29] and L4-100 K rat mAb 6B10 [30]. Cellular protein-specific primary antibodies included polyclonal rabbit Ab raised against the PML protein (Abcam, ab72137) and ß-actin mouse mAb AC-15 (Sigma-Aldrich, Inc.). The used secondary antibodies conjugated to horseradish peroxidase (HRP) for detection by immunoblotting were anti-rabbit IgG, anti-rat IgG and anti-mouse IgG (Jackson/Dianova).

After cell harvesting 24 h p.i., protein extracts were prepared in RIPA lysis buffer, as described elsewhere [31] and separated by SDS-PAGE. Samples were transferred to nitrocellulose blotting membranes (0.45 µm) and proteins were visualized by immunoblotting. *Fiji* [32] was used for quantification analysis and amounts of proteins were calculated relative to actin.

### 2.5. Quantification of Viral DNA

Viral DNA was obtained by proteinase K digestion of protein lysates for 1 h at 55 °C with subsequent enzyme deactivation at 95 °C for 10 min. Quantitative PCR was performed in a CFX Opus 96 Real-Time PCR System (BioRad, Hercules, CA, USA). For this 4 µL of 1:10 diluted DNA, 10 pmol/µL of each forward and reverse oligonucleotide primer, and 5 µL of Luna Universal qPCR Master Mix (NEB) were used together with the following PCR conditions in triplicate: 1 min at 95 °C, 40 cycles of 15 s at 95 °C and 30 s at 63 °C. The viral DNA amounts were calculated in relation to the cellular DNA levels based on the *gapdh* coding region. The oligonucleotides used were as follows: (forward, reverse): Hexon (CGCTGGACATGACTTTTGAG, GAACGGTGTGCGCAGGTA), GAPDH (CATCCTGGGCTACACTGA, TTGACAAAGTGGTCGGTTG).

### 2.6. Transfection of HepaRG Cells

HepaRG cells were seeded in DMEM supplemented with 10% FBS, 100 U/mL of penicillin and 100 µg/mL of streptomycin, 5 µg/mL bovine insulin and 0.5 µM hydrocortisone. 24 h post seeding, cells were treated with DMSO concentrations of up to 1% for 24 h. Then, cells were transfected with 3 µg of pEGFP-C1-expressing eGFP (Clontech) under control of a CMV promoter using linear 25-kDa polyethylenimine (Polysciences, Warrington, PA, USA), as described in (PMID 32184235), for 4 h. Cells were cultured for a further 24 h in medium containing up to 1% DMSO and the number of GFP-expressing cells was determined using a Keyence BZ-X800 fluorescence microscope (Osaka, Japan).

### 2.7. Statistical Analyses

Testing for statistically significant differences in mean values was performed using a *one-way ANOVA* and *Dunnet’s T3* test. All statistical evaluations were performed using the GraphPad Prism10 software version 10.2.0 (San Diego, CA, USA).

## 3. Results

### 3.1. DMSO Increased the Infectability of HepaRG Cells Depending on the Type of Cell-Culture Medium

**Figure 1:**
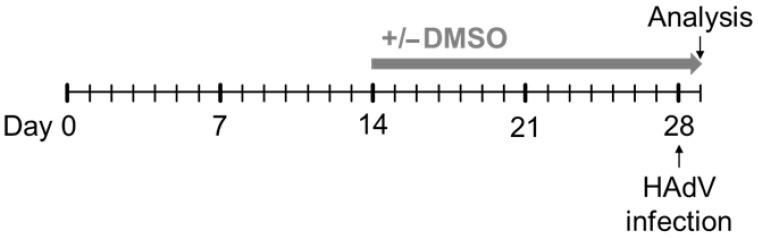
Experimental outline. HepaRG cells were seeded at day 0 in DMEM or WE medium without DMSO. The cells were cultivated for two weeks in medium without DMSO, then, indicated concentrations of DMSO were added to the medium and the cells were cultivated for further two weeks. The medium of the cells was changed every two to three days. After two weeks of culture in medium with or without DMSO, the cells were infected with HAdVs and analyzed 24 h p.i.

To determine if DMSO and its role in differentiation affected HAdV infection of HepaRG cells, DMSO from the manufacturers Carl Roth and SERWA was used in concentrations of 0% and 1% in WE and DMEM medium for infectability analyses. The cells were cultivated in DMEM or WE medium without DMSO for two weeks after seeding. After two weeks, the cells were cultivated for an additional two weeks with or without supplementing the medium with DMSO (Figure 1).

**Figure 2:**
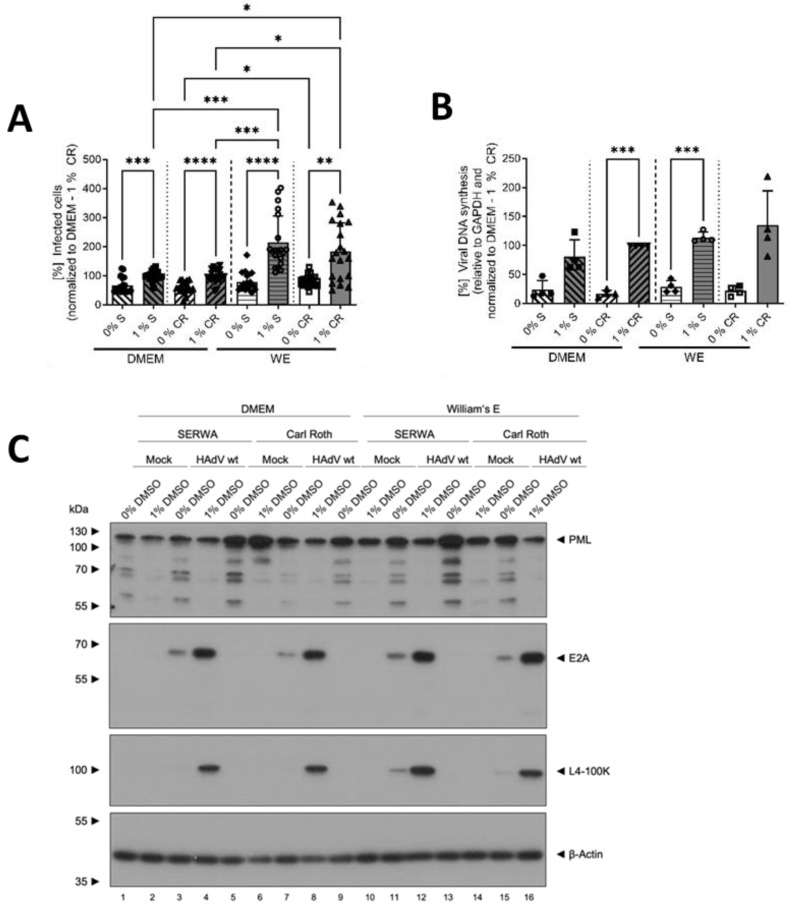

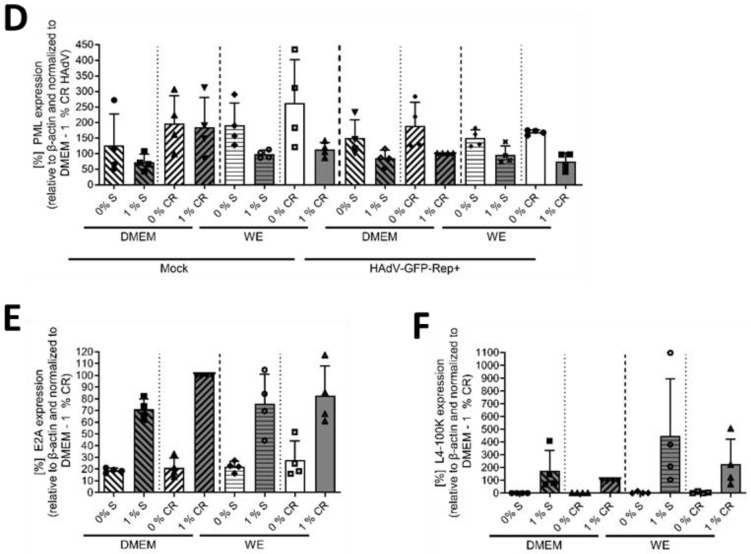
DMSO increased HAdV infection and replication in HepaRG cells. HepaRG cells were differentiated and infected with a replication-competent HAdV-C5 deltaE3 virus, encoding a CMV-promoter-driven eGFP expression cassette, as described in Figure 1, using 0 or 1% DMSO obtained from Serwa (S) or Carl Roth (CR). (**A**) The number of eGFP-expressing, HAdV-infected cells was counted using a Zeiss Axio Observer Z1 and the *Axiovision* software and normalized to HepaRG cells cultivated in DMEM medium containing 1% DMSO obtained from CR. Five view fields were counted per experiment. Bar charts represent mean and standard deviation of four independent biological experiments. At least 1969 infected cells were analyzed. (**B**) Viral DNA synthesis was determined by qPCR relative to GAPDH and normalized to HepaRG cells cultivated in DMEM medium containing 1% DMSO obtained from CR. Bar charts represent mean and standard deviation of four independent biological experiments. (**C**) Cell lysates were prepared and subjected to SDS-PAGE followed by Western blot analysis using mAb B6-8 (E2A), mAb 6B10 (L4-100K), pAb ab72137 (PML) and mAb AC-15 (β-actin). Protein names are depicted on the right, respective molecular weights in kDa on the left of the blot. Data are representative of four independent biological experiments. (**D**–**F**) Protein steady-state levels of PML (**D**), E2A (**E**) and L4-100K (**F**) relative to β-actin were determined by densitometric analysis using *Fiji* and normalized to HAdV-infected HepaRG cells cultivated in DMEM medium containing 1% DMSO obtained from CR. Bar charts represent mean and standard deviation of four independent biological experiments. Statistical significance was determined using a one-way ANOVA and Dunnet’s T3 test. * *p* ≤ 0.05, ** *p* ≤ 0.01, *** *p* ≤ 0.001, **** *p* ≤ 0.0001.

In DMEM, a slightly higher number of HAdV-infected HepaRG cells could be detected for the cells treated with 1% compared to 0% DMSO for both manufacturers. In WE, however, 1% DMSO led to a strong and significant increase in infected cells for both DMSO types compared to 0% DMSO, and even in comparison to 1% DMSO in DMEM medium. These results indicate that DMSO and its role in differentiation led to a higher infectability of HepaRG cells with HAdVs, especially in WE medium. The source of DMSO, however, had no influence (Figure 2A).

To gain further insights into the modulation of HAdV infection by DMSO, viral DNA synthesis and protein expression were determined. The amount of viral DNA quantified by qPCR showed a similar picture, as described above, confirming the results of the infected cell counts. Again, addition of DMSO, independent from the source, led to an increase in viral DNA synthesis and the effect of DMSO was observed to be stronger in WE medium (Figure 2B).

Regarding the protein analyses, PML was chosen as the cellular protein, as it plays an important role during infection with HAdVs [33]. For all conditions, PML amounts decreased non-significantly in the presence of 1% DMSO, whereas no differences were visible for infected versus non-infected cells (Figure 2C,D). As early viral protein, E2A was analyzed, and showed higher steady-state levels in cells treated with 1% DMSO in all conditions (Figure 2C,E). The same result was obtained for the late HAdV protein L4-100K (Figure 2C,F).

Taken together, the data demonstrated that HepaRG cells were more susceptible to HAdV infection and/or better supported HAdV replication in the presence of DMSO. The type of cell-culture medium played an important role, as cells cultivated in WE medium showed significantly stronger effects than those cultivated in DMEM. As the source of DMSO manufacturer had no severe influence on the results outlined above, the following experiments were performed only with DMSO obtained from Carl Roth.

### 3.2. DMSO Dose-Dependently Increased HAdV Infection in HepaRG Cells

**Figure 3:**
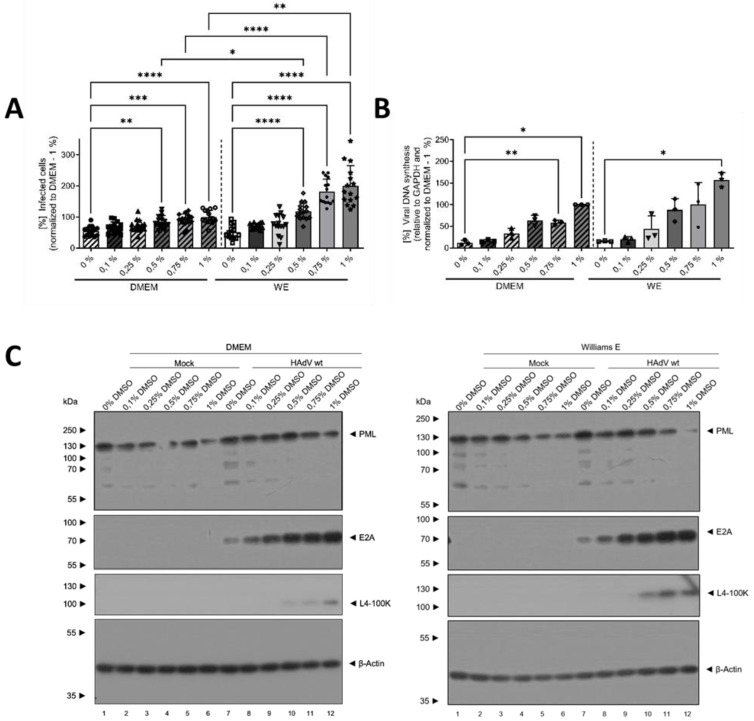

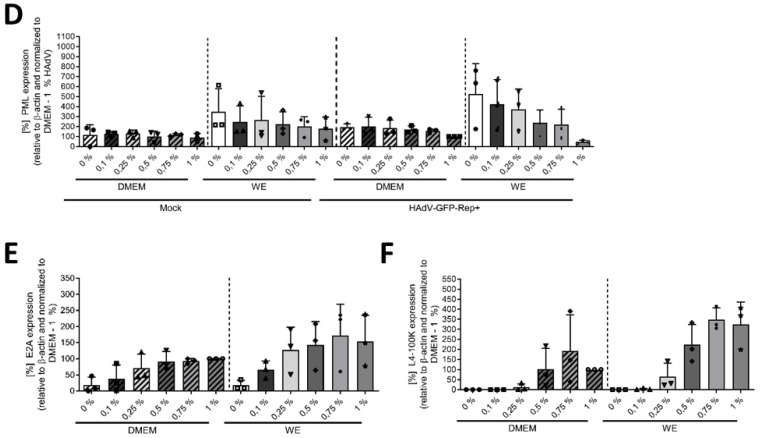
DMSO dose-dependently increased HAdV infection in HepaRG cells. HepaRG cells were differentiated and infected with a replication-competent HAdV-C5 deltaE3 virus, encoding a CMV-promoter-driven eGFP expression cassette, as described in Figure 1, using 0, 0.1, 0.25, 0.5, 0.75 or 1% DMSO obtained from Carl Roth. (**A**) The number of eGFP-expressing, HAdV-infected cells was counted using a Zeiss Axio Observer Z1 and the *Axiovision* software and normalized to HepaRG cells cultivated in DMEM medium containing 1% DMSO. Five view fields were counted per experiment. Bar charts represent mean and standard deviation of three independent biological experiments. At least 2291 infected cells were analyzed. (**B**) Viral DNA synthesis was determined by qPCR relative to GAPDH and normalized to HepaRG cells cultivated in DMEM medium containing 1% DMSO. Bar charts represent mean and standard deviation of three independent biological experiments. (**C**) Cell lysates were prepared and subjected to SDS-PAGE followed by Western blot analysis using mAb B6-8 (E2A), mAb 6B10 (L4-100K), pAb ab72137 (PML) and mAb AC-15 (β-actin). Protein names are depicted on the right, respective molecular weights in kDa on the left of the blot. Data are representative of three independent biological experiments. (**D**–**F**) Protein steady-state levels of PML (**D**), E2A (**E**) and L4-100K (**F**) relative to β-actin were determined by densitometric analysis using *Fiji* and normalized to HAdV-infected HepaRG cells cultivated in DMEM medium containing 1% DMSO. Bar charts represent mean and standard deviation of three independent biological experiments. Statistical significance was determined using a one-way ANOVA and Dunnet’s T3 test. * *p* ≤ 0.05, ** *p* ≤ 0.01, *** *p* ≤ 0.001, **** *p* ≤ 0.0001.

To further characterize the effect of DMSO and its role in differentiation in HAdV infection of HepaRG cells and to determine if there was a dose-dependent effect, cells were treated with different concentrations of DMSO of between 0 and 1% prior to infection with HAdVs, according to the scheme shown in Figure 1. For both WE and DMEM, a linear and dose-dependent increase in the relative number of infected cells with increasing DMSO concentration was observed, showing significantly higher numbers for concentrations ≥ 0.5% DMSO compared to no DMSO treatment. Again, increase was stronger in WE compared to DMEM medium with significantly higher numbers of infected cells at concentrations ≥ 0.5% DMSO (Figure 3A). These findings were confirmed by the amount of viral DNA, which was significantly higher for 0.75% and 1% DMSO in DMEM compared with no DMSO and for 1% DMSO in WE compared to 0% DMSO (Figure 3B). The results of protein analyses showed a decrease in PML with increasing DMSO concentrations in the HAdV-infected cells for both media, while in uninfected cells, the effect was only observed slightly for WE medium (Figure 3C,D). E2A amounts increased with increasing DMSO concentrations. In WE and DMEM, the steady-state levels reached a plateau at 0.5% DMSO (Figure 3C,E). The amounts of L4-100 K showed a peak at 0.75% DMSO for both media types (Figure 3C,F). Adenoviral protein levels were in general higher in cells cultivated in WE (Figure 3C,E,F). Taken together, DMSO treatment led to a dose-dependent increase in HAdV infection, replication and protein expression which was more pronounced in HepaRG cells cultivated in WE compared with DMEM medium.

### 3.3. The Mechanism of Action of DMSO Was Based on Viral Entry and Not on Viral Replication

**Figure 4:**
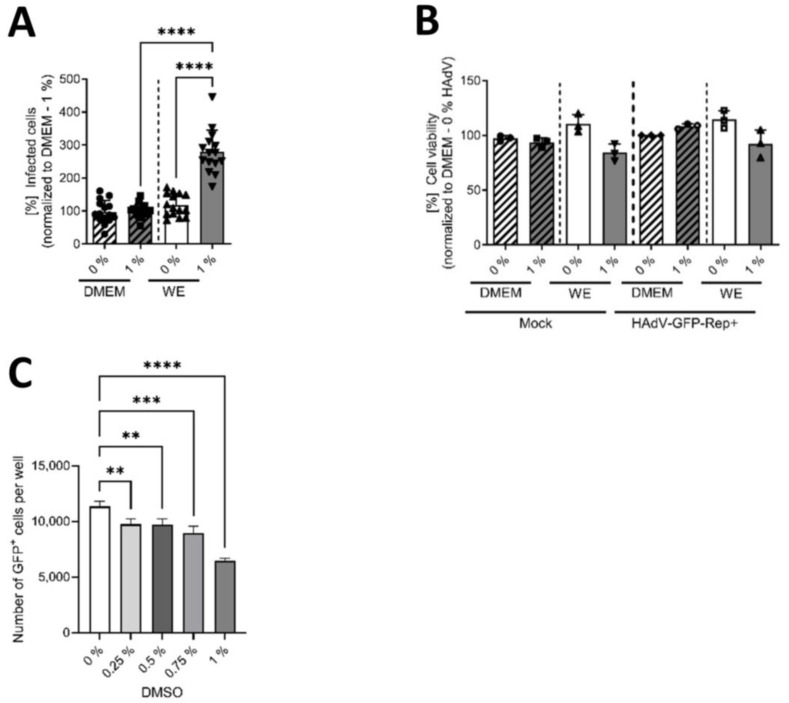
DMSO enhanced the susceptibility of HepaRG cells to viral entry in WE medium. HepaRG cells were differentiated and infected with a replication-deficient, eGFP-expressing HAdV-C5-based first-generation adenoviral vector, as described in Figure 1, using 0 or 1% DMSO obtained from Carl Roth. (**A**) The number of eGFP-expressing cells that absorbed the replication-deficient HAdV-based vector was counted using a Zeiss Axio Observer Z1 and the *Axiovision* software and normalized to HepaRG cells cultivated in DMEM medium containing 1% DMSO. Five view fields were counted per experiment. Bar charts represent mean and standard deviation of three independent biological experiments. At least 1057 infected cells were analyzed. Statistical significance was determined using a one-way ANOVA and Dunnet’s T3 test. **** *p* ≤ 0.0001. (**B**) Cell viability was determined of infected (with a replication-competent HAdV-C5 deltaE3 virus, encoding a CMV-promoter-driven eGFP expression cassette) and non-infected (Mock) cells using the Promega CellTiter-Blue^®^ Cell Viability Assay system. (**C**) HepaRG cells were seeded and, after 24 h, treated with DMSO at concentrations of up to 1% for a further 24 h before transfection with pEGFP-C1 expressing eGFP under control of a CMV promoter for 4 h. After transfection, cells were again cultured in medium containing up to 1% DMSO. GFP-positive cells were determined at 24 h post transfection by fluorescence microscopy. Statistical significance was determined using a one-way ANOVA and Dunnet’s T3 test. ** *p* ≤ 0.01, *** *p* ≤ 0.001, **** *p* ≤ 0.0001. Bar charts represent mean and standard deviation of three independent biological experiments.

The final experiments were performed to obtain information on the mechanism of action of DMSO concerning the higher rate of HAdV infection of HepaRG cells. By using a replication-deficient, eGFP-expressing HAdV-based vector, we investigated whether the increase in HAdV infection and replication mediated by DMSO, and its support of differentiation, was due to enhanced viral replication or viral entry. Figure 4A shows that in the case of WE medium, significantly higher numbers of infected cells were observed in cells cultivated with 1% DMSO compared with non-treated cells. In DMEM, no differences could be found and numbers of infected cells were significantly lower compared with cells cultivated in WE with 1% DMSO. To determine if DMSO and/or HAdV infection affect HepaRG cell viability, a CTB assay was performed for cells treated with 0 or 1% DMSO. No toxicity of DMSO could be observed with either media type, as cell viabilities did not show significant differences between the conditions (Figure 4B). To further validate that the increase in eGFP-expressing, HAdV-infected cells in the samples treated with DMSO was not due to an increase in CMV-promoter activity, HepaRG cells were treated with DMSO concentrations of up to 1%, transfected with a plasmid expressing eGFP under the control of the CMV promoter and the number of GFP-positive cells was determined 24 h post transfection. Here, we could even observe a decrease in the number of GFP-expressing cells in the samples treated with DMSO, substantiating the finding that DMSO treatment induced HAdV infectivity.

These results demonstrated that DMSO and DMSO-mediated modulation of differentiation enhanced viral entry into HepaRG cells by affecting their susceptibility to viral infection and that this effect was mostly present in WE medium, and not in DMEM medium.

## 4. Discussion

The present work demonstrated that DMSO, and its role in differentiation, increased infectability of HepaRG cells with HAdVs dependent on the used medium for cell cultivation. William’s E medium, which is commonly chosen for the cultivation of hepatocytes, led to a stronger increase in infectability of HepaRG cells when supplemented with DMSO than DMEM medium. Consequently, differences in the media compositions contributed to the observed effects. In Table S1, the ingredients of WE and DMEM are listed, showing that WE contains numerous additives which are not included in DMEM, especially vitamins and amino acids. DMEM, however, includes more than double the amount of glucose. Most of the components present in both media differ in their concentrations. Mörk et al. compared these culture media for analyses of bile-acid transport in primary human hepatocytes and could also find media-dependent differences. Cells cultivated in WE showed stronger uptake and excretion of taurocholate compared with cells cultivated in DMEM. The authors could demonstrate that this was due to higher mRNA expression for bile-acid-specific transporter proteins in cells cultivated in WE. They assumed that higher levels of glutathione or ascorbic acid in WE might be the reason. However, supplementation of DMEM did not lead to comparable taurocholate transport [34]. Additionally, hepatocytes cultivated in DMEM exhibited fewer and smaller bile canaliculi, which was supported by the results of Chandra et al. [34,35]. Potentially, DMSO uptake was higher in cells cultivated in WE medium due to stronger membrane transportation processes, which should be investigated in future research.

In vivo, HAdVs mainly infect terminally differentiated cells. This is due to the fact that the differentiation status of the host cells affects and enhances the activity of early adenoviral promoters and therefore influences effective viral DNA replication [36,37,38,39,40,41]. As DMSO enhances and supports differentiation of HepaRG cells and hepatic cells in general [4,12,14,15,16], the increase in HAdV infectability mediated by DMSO might be attributed to a more robust differentiation of the HepaRG cells. Nevertheless, DMSO could also have further impact on the cells, in addition to a role in the support of differentiation. The enhanced infectability in the presence of DMSO might be the result of its impact on cell-membrane permeability and elasticity [42], which might alter the mechanisms of viral uptake into the cell. In addition, the anti-inflammatory effects, which are frequently reported for different cell types [4,11,43,44], might also facilitate viral infection. In Caco-2 cells, IL-6 and MCP-1 secretions were reduced dose-dependently by DMSO [45] while in whole human blood samples, it significantly suppressed the expression of pro-inflammatory chemokines, cytokines and prostaglandin E2 [44]. HepaRG cells exposed to 2% DMSO showed a set of 288 downregulated genes, which had anti-inflammatory upstream regulators like cytokine tumor necrosis factor (TNF) and oncostatin M as well as lipopolysaccharide, in a work by Dubois-Pot-Schneider et al. [4]. It is therefore tempting to speculate that HepaRG cells in the present study were more susceptible to infections with HAdVs because DMSO mediated downregulation of the production of and the reaction pathways to pro-inflammatory chemokines and cytokines, that normally activate cell-intrinsic and cell-extrinsic immune defense programs and mediate cell–cell communication [46]. However, based on our findings we also hypothesize that rather, DMSO affects at the level of virus entry. Thus, we need to also highlight the possible effect of virus infection-induced inflammatory cytokines on bystander cells and, simultaneously, on a potential second round of viral infection events. As we did use a very low moi and we did not induce synchronized infection processes, we might also speculate that only cells infected early can trigger an IFN response and thus prime bystander cells, which might be blocked during DMSO treatment.

Interestingly, one of the downregulated genes reported in the work of Dubois-Pot-Schneider was coding for PML [4]. This is in concordance with the findings of the present work, where a reduction in PML protein levels was observed with increasing DMSO concentrations. As reported recently, different PML isoforms have either beneficial or repressive functions concerning HAdV replication [47]. It might be interesting to investigate whether the expression of HAdV-repressive isoforms is specifically downregulated by DMSO, which might contribute to an enhanced adenoviral infectivity.

## 5. Conclusions

Taken together, our data demonstrate that the used medium for cell culture may have a strong influence on performed experiments. It should carefully be considered, which medium suits best the used cell type and performed analyses and this should also be taken into account when comparing results with data from other research groups. Additionally, we could show that DMSO and DMSO-induced support of HepaRG differentiation had a strong effect on viral infectivity, as it enhanced entry of HAdVs into HepaRG cells. As DMSO is often used as solvent for potential antiviral drugs, it must be ensured that the observed effects in those studies are not biased by DMSO. Also, DMSO-dependent effects must be considered when it is used as cryopreservant and it should be guaranteed that it is removed completely when seeding cells. In accordance with results of other research groups [11,48], this work emphasizes that DMSO, e.g., acting as a stabilizer of differentiation in HepaRG cells, is not inert in cell cultures.

## Data Availability

Data is contained within the article or Supplementary Material.

## Supplementary Materials

The following supporting information can be downloaded at: https://www.mdpi.com/article/10.3390/v16040633/s1, Table S1: Comparison of the composition of William’s E and DMEM medium.
