# Supplementary figures and images for: DMSO and Its Role in Differentiation Impact Efficacy of Human Adenovirus (HAdV) Infection in HepaRG Cells
Source: Viruses. 2024 Apr 19;16(4):633. doi: 10.3390/v16040633 (PMC11054035; doi:10.3390/v16040633)

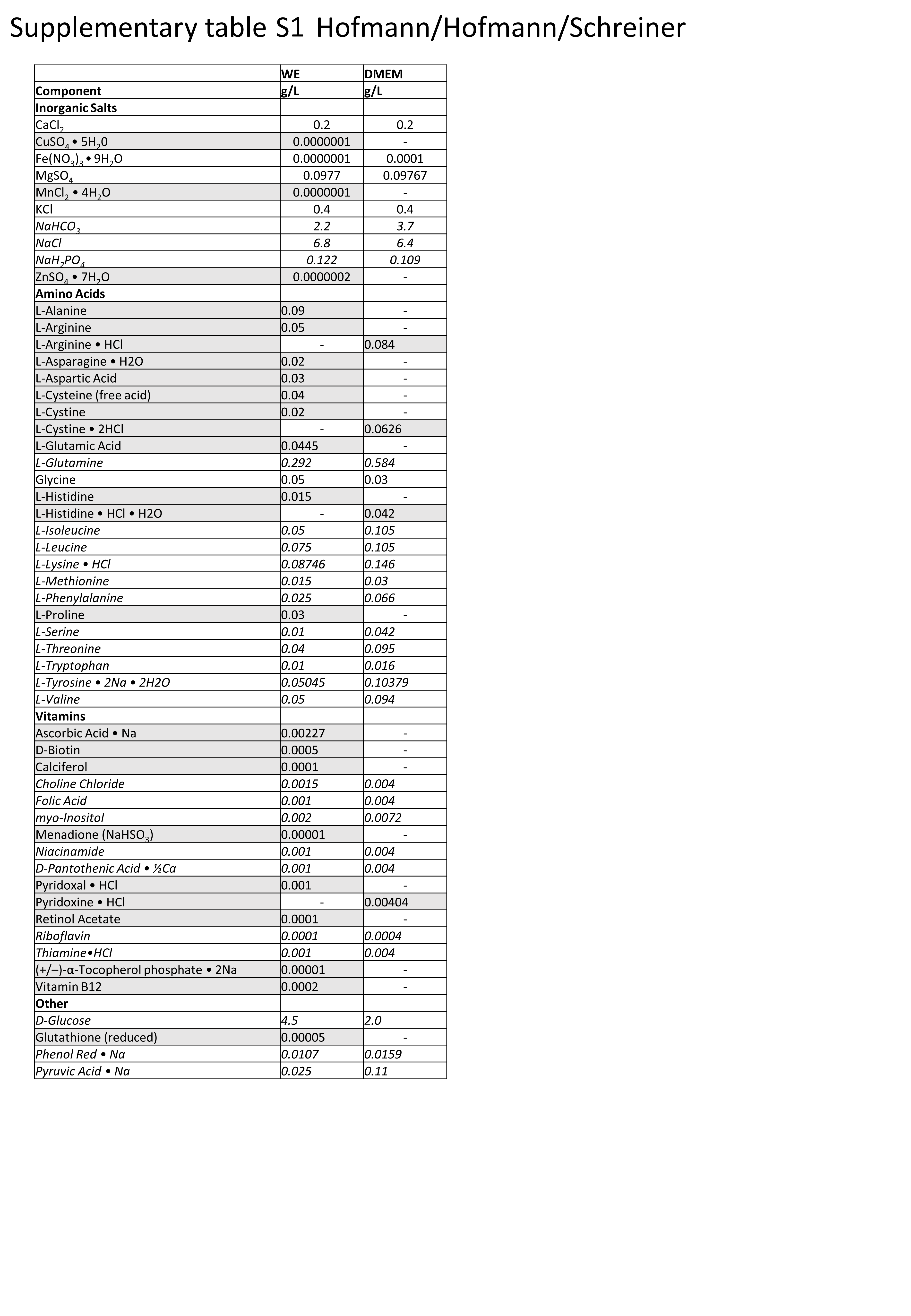

Supplement: Supplementary file 1 [file viruses-16-00633-s001.zip › viruses-2941545-supplementary.TIF]
